# Comparative Lactylomic Profiling Unveils Dynamic Reprogramming of Post-translational Lactylation Landscapes During Human Sperm Capacitation

**DOI:** 10.1016/j.mcpro.2026.101657

**Published:** 2026-09-08

**Authors:** Guohui Zhang, Yuhong Zhao, Yu Lei, Weiwei Zhi, Wenming Xu, Jiuzhi Zeng, Li Wang, Fangyi Long, Juncen Guo, Weixin Liu

**Affiliations:** 1 Center of Women’ s and Children’ s Health Medical Research and Technology Innovation, Sichuan Provincial Women’ s and Children’ s Hospital/The Afffliated Women’ s and Children’ s Hospital of Chengdu Medical College, Chengdu Medical College, Chengdu, China; 2 Key Laboratory of Reproductive Medicine, Sichuan Provincial Woman’ s and Children’ s Hospital, The Affiliated Women’ s and Children’ s Hospital of Chengdu Medical College, Chengdu, China; 3 Department of Obstetrics and Gynecology, Joint Laboratory of Reproductive Medicine (SCU-CUHK), Key Laboratory of Obstetric, Gynecologic and Pediatric Diseases and Birth Defects of Ministry of Education, West China Second University Hospital, Sichuan University, Chengdu, China; 4 School of Clinical Laboratory Medicine, Chengdu Medical College, Chengdu, China

**Keywords:** sperm capacitation, lactylation, metabolic reprogramming, energy metabolism, microtubule dynamics

## Abstract

Lactylation, a novel post-translational modification involving lactate conjugation to lysine residues, bridges metabolic flux and epigenetic regulation and has emerged as a conserved regulatory mechanism across eukaryotes and prokaryotes, governing cell proliferation, immune responses, and metabolic reprogramming via a “Writer-Eraser” system. Despite the established roles of lactylation in energy metabolism, its function in male reproduction remains unexplored. This study investigates the lactylation modification dynamics during human sperm capacitation, a process requiring metabolic reprogramming for hyperactivated motility. Through 4D FastDIA-based quantitative dynamic profiling, we first conducted the comprehensive characterization of lysine lactylation modifications during sperm capacitation, systematically mapping differential lactylation patterns at the pathway, proteomic, and site-specific levels. We identified 2030 quantifiable lactylated proteins, revealing significant capacitation-dependent modulation: 115 proteins with 133 sites demonstrated upregulated lactylation, while 287 proteins containing 471 sites showed downregulation (*p* < 0.05, Fold Change >1.5). Notably, 30 proteins exhibited bidirectional regulatory patterns with coexisting upregulated and downregulated sites, and a predominant proportion of modified proteins (263 of 372) displayed single-site differential lactylation. A marked reduction in lactylation levels of mitochondrial respiratory chain proteins was observed after sperm capacitation, indicating heightened ATP requirements. Moreover, proteins with differential lactylation were enriched in biological processes including fertilization, sperm capacitation, β-oxidation, and microtubule dynamics. Strikingly, the presence of known lactylation regulatory enzymes including Alanine–tRNA ligase 1 (AARS1), Sirtuin (SIRT) 2/3, and Histone deacetylase (HDAC) 1/2/6 were detected in the sperm proteome, revealing a 1.6-fold upregulation of SIRT3, implicating its role in delactylation regulation during capacitation. Collectively, these findings delineate a lactylation-coordinated mechanism governing mitochondrial oxidative phosphorylation optimization and cytoskeletal architecture remodeling. This work substantiates a trinity regulatory mechanism integrating "metabolism-epigenetics-function" axes through lactylation circuitry, which may have implications for understanding male infertility.

## Highlights

• First comprehensive lactylome landscape of human sperm capacitation.
• SIRT3 is a potential regulator of capacitation-associated delactylation.
• Honokiol reduces lactylation and enhances sperm motility during capacitation.

## Graphical abstract

**Figure:**
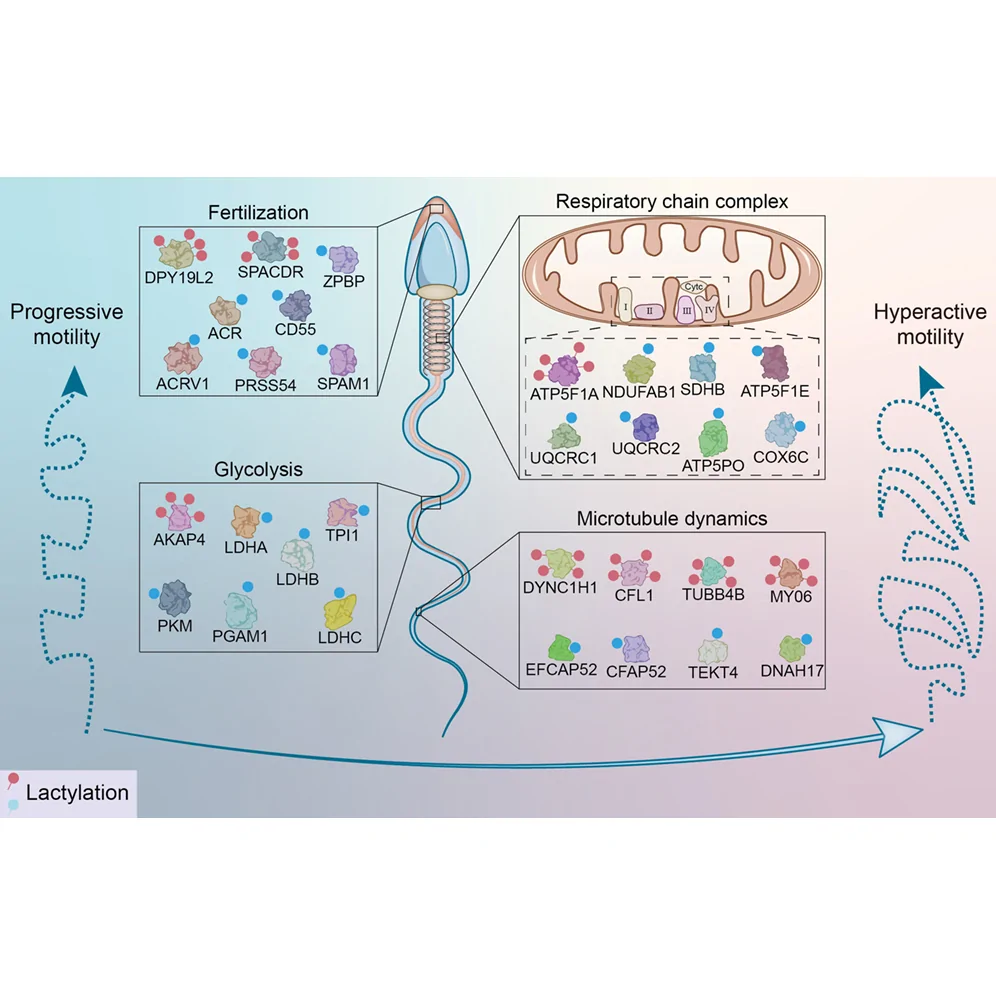

## In Brief

Protein lactylation is a newly discovered modification linking metabolism to cellular function, but its role in male reproduction remains unknown. Here, we present the first comprehensive lactylome landscape of human spermatozoa during capacitation. Our findings reveal a widespread decline in lactylation on mitochondrial respiratory chain proteins, accompanied by the presence of key lactylation regulators in sperm. This work uncovers a potential lactylation-coordinated mechanism governing energy metabolism and sperm function, providing a foundation for understanding male infertility at the post-translational modification level.

Lactylation, an emerging post-translational modification (PTM), is characterized by the enzymatic conjugation of lactate to lysine residues through covalent bonds. This groundbreaking discovery has established a novel molecular bridge connecting metabolic intermediates with epigenetic regulation while offering transformative perspectives for understanding the interplay between energy metabolism and disease etiology. The field witnessed a paradigm shift in 2019 when Professor Yingming Zhao’s seminal work published in Nature unveiled histone lactylation modification, fundamentally redefining lactate’s biological significance from a mere metabolic by-product to a bona fide epigenetic modulator (1). The research frontier has since expanded exponentially beyond histone modifications, with recent advances confirming the phylogenetically conserved nature of lactylation across eukaryotic and prokaryotic species (2). Cumulative evidence from 2019 to present has systematically elucidated lactylation’ s master regulatory functions in orchestrating critical physiological processes including cellular homeostasis (proliferation/differentiation/apoptosis), inflammatory cascades, immune surveillance networks, and metabolic reprogramming (2, 3, 4).

The homeostatic control of lactylation is governed by an intricate "Writer-Eraser" enzymatic coordination system: The Writer machinery, comprising acyltransferases like CBP/p300, facilitates lactyl moiety transfer from lactyl-CoA donors to specific lysine residues (1), while the Eraser system, predominantly class I histone deacetylase isoforms (HDAC1-3), executes precise delactylation through catalytic removal of lactyl groups to ensure dynamic reversibility (5). This biochemical equilibrium is further modulated by microenvironmental determinants including lactate gradients (via monocarboxylate transporters), glycolytic activity, and NAD+/NADH redox status (6, 7). Functioning as a multimodal regulatory nexus, lactylation modification forms a complex regulatory network by integrating metabolic, epigenetic, and signaling pathways. Pathologically, lactylation aberrations manifest as disease-specific molecular signatures: Oncogenic lactylation in tumor niches stabilizes DNA repair proteins through site-specific modifications, conferring therapeutic resistance via enhanced genomic stability (8, 9). In pulmonary sepsis pathogenesis, H3K18 lactylation-driven Methyltransferase-like 3 (METTL3) upregulation induces ferroptosis in alveolar epithelium (10). In neurodegenerative diseases, lactylation modification exacerbates neuroinflammation in Alzheimer’ s disease by regulating microglial metabolic reprogramming (11). Lactylation modification finely regulates the dynamic equilibrium between glycolysis and oxidative phosphorylation (OXPHOS) through a “metabolism-epigenetics-signal transduction” trinity mechanism. Its core lies in the dual role of lactate: both as an energy metabolism product and as a signaling molecule that modulates metabolic networks. By modifying the functions of enzymes and transcription factors (TFs), lactylation enables cells to rapidly adapt to microenvironmental changes. Structural studies of pyruvate kinase M2 (PKM2) demonstrate lactylation-induced stabilization of the active tetrameric conformation, which reduces glycolytic flux by shunting pyruvate into mitochondria for OXPHOS, promoting their transition to a reparative phenotype. Contrastingly, aldolase A (ALDOA) lactylation in hepatic cancer stem cells promotes glycolysis flux through disrupted dead box deconjugate enzyme 17 (DDX17) binding, maintaining its stemness (12). Muscle-specific analyses reveal that L-lactate dehydrogenase A chain (LDHA)-derived lactylation suppresses electron transport chain (ETC) complex IV activity, creating an energy sensor system that adapts mitochondrial output to glycolytic output (13). Despite these advances, the reproductive dimension of lactylation biology, particularly its potential role in spermatogenic metabolism and testicular energy homeostasis, remains conspicuously absent from current research paradigms.

Within the female reproductive tract, sperm acquire fertilization competence through a series of well-defined physiological changes collectively termed “capacitation,” encompassing membrane destabilization (acrosome reaction) and a transition from linear progression to hyperactivated flagellar asymmetric beating, both of which facilitate sperm navigation and oocyte fertilization (14, 15). The hyperactivated state is characterized by rapid asymmetric flagellar oscillations requiring substantial ATP supply. Recent research by Cameron A. Schmidt *et al*. (16) demonstrates that murine sperm dynamically redistribute metabolic flux in response to microenvironmental cues to achieve hyperactivation. Their findings reveal pyruvate as a critical microenvironmental metabolite that rapidly elevates the NAD+/NADH redox ratio via LDHC activity, concurrently accelerating glycolytic flux in the flagellar principal piece to promote lactate production while suppressing mitochondrial lactate oxidation, thereby programming sperm for hyperactivation. Notably, recent studies show hypoxia-induced mitochondrial protein lactylation suppresses OXPHOS and ATP synthesis (17). Given lactate’ s dual role as both metabolic fuel and lactylation mediator during capacitation, we hypothesize functional protein lactylation may critically regulate this process. Therefore, we performed lactylation modification profiling of human sperm before and after *in vitro* capacitation. Intriguingly, post-capacitation sperm exhibited significantly reduced lactylation levels on mitochondrial respiratory chain complex proteins, aligning with increased ATP demands during capacitation, suggesting delactylation induced enzymatic activity elevation. Moreover, proteins with differential lactylation were enriched in biological processes (BP) including fertilization, fatty acid β-oxidation, RNA splicing/translation, protein degradation, molecular chaperone processing, and microtubule dynamics. Particularly noteworthy is flagellar tubulin lactylation, while PTMs like glutamylation (18) and glycylation (19) are known to regulate flagellar motility, tubulin lactylation’ s functional role in sperm hyperactivation represents a novel discovery with significant scientific implications. Our study systematically maps functional protein lactylation dynamics during capacitation, identifies key modified pathways/proteins, and characterizes specific lactylation sites. These findings propose a novel mitochondrial energy regulation mechanism through lactylation, offering innovative perspectives for understanding capacitation.

## Materials and Methods

### Study Participants

The patients included in this study were sourced from the Sichuan Provincial Woman’ s and Children’ s Hospital. Semen samples were obtained through patient masturbation. The patients were 20 to 40 years of age; moreover, any other diseases or abnormalities that could induce infertility were eliminated, including radio-chemotherapy, varicocele, inflammation of the testis and epididymis, mumps, congenital factors, and endocrine abnormalities. Semen samples were collected from healthy donors in accordance with the World Health Organization (WHO) guidelines (sixth edition). Only specimens with normal sperm morphology and progressive motility ≥32% were included. The study was performed in accordance with the guidelines of the Declaration of Helsinki and approved by the Medical Ethics Committee of Sichuan Provincial Woman’ s and Children’ s Hospital (20,240,903–357), and all patients provided signed informed consents.

### Sperm Collection and Processing

Following liquefaction at 37°C, the samples were washed using Grad Medium (C009, AIVFO) to remove seminal plasma. The processed sperm suspensions were equally divided into two aliquots. The first aliquot was immediately snap-frozen in liquid nitrogen and stored at −80°C as the pre-capacitation sperm sample. The second aliquot was resuspended in TYH capacitation medium (M2035, Nanjing Aibei Biotechnology), which had been pre-equilibrated overnight in a 37°C incubator with 5% CO_2_. Capacitation was induced by incubating the suspension under the same conditions (37°C, 5% CO_2_) for 2 h. Post-capacitation, the spermatozoa were snap-frozen in liquid nitrogen and transferred to −80°C for long-term storage as the post-capacitation sperm sample. Both pre- and post-capacitation samples were reserved for subsequent Western blot and lactylation proteomics analyses. N = 3 in pre- and post-capacitation group for lactylation proteomics analyses, each sample was obtained by mixing samples from 10 individuals.

For semen analysis, semen samples were collected and placed at 37°C for 30 to 60 min to allow complete liquefaction. An aliquot of 3 to 5 μl of fully mixed liquefied semen was loaded onto a disposable sperm counting chamber (SC-IV) and analyzed using a SAS fully automated semen analyzer (model SAS-A100SE; SAS Medical Technology) equipped with the SAS sperm analysis system (version SP2). The system utilizes computer-aided sperm analysis (CASA) technology to automatically capture dynamic images of spermatozoa from multiple fields and assess sperm concentration, total motility, progressive motility, and kinematic parameters. All procedures were performed in accordance with the World Health Organization Laboratory Manual for the Examination and Processing of Human Semen.

### Experimental Design and Statistical Rationale

The study employed three independent biological replicates for both the pre- and post-capacitation groups. Importantly, to minimize confounding effects from interindividual biological variation, each biological replicate was generated by pooling semen samples from 10 distinct healthy donors, resulting in a total of 30 donors. This robust pooling strategy effectively normalizes donor-specific baseline noise, thereby substantially enhancing the statistical validity of the observed capacitation-induced changes. Given the inherently high quantitative stability and low missing-value rate of the modern dia-PASEF (data-independent acquisition) mass spectrometry approach, a single technical injection was performed per biological replicate. Strict quality control parameters were applied, with protein and peptide false discovery rates (FDRs) controlled below 1%. We further justify that this sample size, combined with the pooling strategy, provides sufficient statistical power for robust evaluation using Student’s t-tests (*p* < 0.05, fold change >1.5 or < 0.67).

### Protein Extraction

The samples were retrieved from storage at −80°C. Subsequently, a mixture comprising four volumes of lysis buffer (containing 1% SDS, 1% protease inhibitor cocktail, and 3 μM TSA and 50 mM NAM) was introduced to the cell sediment. This was followed by sonication for 3 minutes on ice, utilizing a high-intensity ultrasonic processor (Scientz). The residual debris was then eliminated by centrifugation at 12,000 g for 10 min at 4°C. Ultimately, the supernatant was harvested, and the protein concentration was quantified using the BCA kit (23,227, Thermo), following the manufacturer’ s protocol.

### Protein Preprocessing

The protein sample was initially added with one volume of pre-cooled acetone and vortexed thoroughly. Subsequently, an additional four volumes of pre-cooled acetone were added, and the mixture was precipitated at −20 °C for 2 h. The resulting precipitate was washed 2 to 3 times with pre-cooled acetone. Afterward, the protein was redissolved in 200 mM TEAB and dispersed using ultrasonication. Trypsin was added at a mass ratio of 1:50 (trypsin-to-protein) for an overnight digestion. The sample was then reduced with 5 mM dithiothreitol for 60 min at 37 °C and alkylated with 11 mM iodoacetamide for 45 min at room temperature in the dark. Finally, the peptides were purified via desalting using a Strata X SPE column.

### Pan Antibody-Based PTM Enrichment

Lactylated peptides were enriched using Anti-L-lactyllysine Antibody Conjugated Agarose Beads (Catalog No. PTM-1404, PTM Bio). According to the product datasheet, this recombinant rabbit monoclonal antibody was raised against L-lactylated lysine peptides as the immunogen and specifically recognizes L-lactyllysine residues without cross-reactivity with other structurally similar modifications such as D-lactylated or acetylated lysine residues. The antibody is covalently immobilized onto agarose beads (4 mg/ml immobilized antibody, supplied as a 50% slurry containing 66% glycerol). For enrichment, tryptic peptides (2 mg per sample) were dissolved in NETN buffer (100 mM NaCl, 1 mM EDTA, 50 mM Tris-HCl, 0.5% NP-40, pH 8.0) and centrifuged at 12,000*g* for 10 min at 4 °C to remove insoluble materials. Aliquots of 40 μl bead slurry were washed twice with pre-chilled PBS and then incubated with the peptide solution overnight at 4 °C with gentle end-over-end rotation. After incubation, the beads were collected by centrifugation at 1000*g* for 1 min and washed four times with NETN buffer and twice with water. Bound lactylated peptides were eluted twice with 0.1% trifluoroacetic acid (15 min incubation each at room temperature). The eluates were combined and vacuum-dried. Because the antibody is covalently conjugated to the beads, the beads are separated by centrifugation after elution, and no free antibody is present in the final sample submitted for mass spectrometry analysis. The dried peptides were desalted using C18 ZipTips (Millipore) prior to LC-MS/MS analysis.

### LC-MS/MS Analysis

The peptides were dissolved in solvent A (0.1% formic acid, 2% acetonitrile in water) and directly loaded onto a homemade reversed-phase analytical column (25 cm × 100 μm i.d.). The mobile phase consisted of solvent A and solvent B (0.1% formic acid in acetonitrile). Peptides were separated using the following gradient: 6% to 22% B over 0 to 18 min, 22%-30% B over 18 to 22 min, 30% to 80% B over 22 to 26 min, and 80% B over 26 to 30 min, at a constant flow rate of 450 nl/min using a NanoElute UHPLC system (Bruker Daltonics). The peptides were then analyzed by capillary electrospray ionization followed by timsTOF Pro 2 mass spectrometry, with an applied voltage of 1.65 kV. The instrument was operated in data-independent parallel accumulation serial fragmentation (dia-PASEF) mode. The full MS scan range was set to 100 to 1700 m/z, and 8 PASEF-MS/MS scans per cycle were acquired. The MS/MS scan range was set to 425 to 1025 m/z with an isolation window of 25 m/z.

### Data Processing

The DIA data were processed using Spectronaut (v.18) software. The tandem mass spectra were searched against the UniProtKB/Swiss-Prot human reference proteome database (Homo_sapiens_9606_SP_20231220.fasta), containing exactly 20,429 protein sequence entries. For dia-PASEF data acquired on a timsTOF Pro two system, both precursor and fragment ion mass tolerances were set to "Dynamic" (automatic) in Spectronaut. Trypsin/P was specified as the cleavage enzyme, allowing up to four missed cleavages (due to potential reduced cleavage efficiency after lactylation modification). Carbamidomethylation on cysteine was set as a fixed modification; acetylation on protein N-termini, oxidation on methionine, and lactylation on lysine (C_3_H_4_O_2_, +72.02 Da) were set as variable modifications. Peptide confidence was assessed using the Posterior Error Probability (PEP) calculated by Spectronaut. Only peptides with a PEP ≤0.01 (corresponding to a 1% FDR at the peptide level) were retained for further analysis. Protein sequence coverage was calculated as the percentage of the protein sequence covered by all identified unique peptides. The FDR for proteins, peptides, and peptide-spectrum matches was controlled at < 1%.

### Clustering and Functional Annotation of Differentially Modified/Expressed Proteins

We set the differential threshold of foldchange 1.5 and adjusted *p*-value 0.05 for significantly different modified sites and different expression proteins. Protein clusters were generated based on differential lactylation or expression patterns across experimental groups using hierarchical clustering (R packages hclust and cutree). Expression dynamics within clusters were visualized by plotting mean protein abundance with 95% confidence intervals (calculated via *t* test) across conditions. Functional enrichment analysis of Gene Ontology (GO) terms was performed for each protein cluster using clusterProfiler (v3.2.14). GO annotations were retrieved from the UniProt-GOA database (http://www.ebi.ac.uk/GOA/). Protein identifiers were mapped to UniProt IDs prior to GO term assignment. For proteins lacking UniProt-GOA annotations, InterProScan was implemented to predict GO functions through domain-specific sequence alignment. Protein-protein interactions were analyzed in STRING (https://string-db.org/). For volcano plots, *p* values were calculated by Student’s t-tests. All boxplots and bubble plots were generated using the R package “ggplot2”.

### Protein Structure Prediction and Protein–Protein Docking

Three-dimensional structural models of SIRT3, Citrate synthase (CS), Voltage-dependent anion-selective channel protein 1 (VDAC1), ATP synthase peripheral stalk subunit OSCP (ATP5PO), ATP synthase F(1) complex subunit alpha (ATP5F1A), Enolase 1 (ENO1) and PKM were downloaded from the Alpha Fold Protein Structure Database (20, 21) (https://alphafold.com/). Flexible docking between SIRT3 and each of its predicted modified proteins were performed on the HADDOCK webserver https://rascar.science.uu.nl/haddock2.4/) (22). The first complex model with the lowest HADDOCK score was chosen to be the final model. The structure diagram was made by PyMOL software.

### Co-Immunoprecipitation (Co-IP)

The extracted total proteins from the sperm were incubated with 3 to 5 μl target antibodies, including Anti-L-Lactyl Lysine (PTM Bio, PTM-1404), SIRT3 (Abclonal, A7307), CS (Proteintech, 16131-1-AP), ATP5F1A (Proteintech, 14676-1-AP), ENO1 (Proteintech, 11204-1-AP), PKM (Proteintech, 15822-1-AP), Succinate dehydrogenase [ubiquinone] iron-sulfur subunit (SDHB) (Proteintech, 10620-1-AP), cytochrome c oxidase subunit 6C (COX6C) (Santa, sc-390414), and Cytochrome b-c1 complex subunit 1 (UQCRC1) (Proteintech, 21705-1-AP), on the rotary shaker overnight at 4 °C. Subsequently, 50 μl of Protein A/G magnetic beads (Thermo Fisher, 88,804) were added into each sample and incubated for 60 min at room temperature. After washing twice and resuspension in PBS, the proteins were eluted from the beads by denaturation in 1xSDS (Biosharp, BC502 A) at 95 °C for 15 min. The obtained proteins were used for further Western blot analysis.

### Western Blot

The sperm were lysed on ice in RIPA lysis buffer for 60 min (Beyotime, P0013 B), which contained the Protease Inhibitor Cocktail (Biomake, B14012). Subsequently, protein concentrations of each sample were determined by the BCA method (Solarbio, PC0020). Next, the same amounts of total proteins from 30 to 60 μg were loaded into each sampling hole and were separated via electrophoresis by 7.5 to 12.5% SDS-PAGE (Lablead, P41212) and then transferred to polyvinylidene difluoride membranes (Millipore, ISEQ00010). Subsequently, the membranes were successively incubated with primary antibodies overnight at 4 °C and secondary antibodies for 60 min at room temperature. Finally, the protein bands were visualized with ECL HRP substrate (Lablead, E1060). β-actin and α-Tubulin were used as the loading control to ensure that the total protein contents was consistent in each sampling well. Finally, ImageJ was used for grayscale value analysis. The following primary and secondary antibodies were used: SIRT3 (Abclonal, A7307, 1:1000), VDAC1 (Proteintech, 10701-1-AP, 1:2000), α-Tubulin (Abclonal, A6830, 1:2000), β-actin (Abclonal, A19056, 1:10,000), ATP5O (PCM, PC-68211, 1:1000), Acyl carrier protein (NDUFAB1) (Abclonal, A21114, 1:2000), ATP synthase F(0) complex subunit g (ATP5L) (Abclonal, A9178, 1:1000), Anti-L-Lactyl Lysine (PTM Bio, PTM-1404, 1:1000), Anti-Phosphotyrosine (PY20) (Santa, sc-508, 1:500), LDHA (Abclonal, A28205, 1:1000), Malate dehydrogenase (MDH2) (Abclonal, A13516, 1:1000), ODF1 (Santa, sc-390152, 1:500), HRP-conjugated Donkey anti-Mouse IgG (H + L) (Abclonal, AS033, 1:5000), HRP-conjugated Goat anti-Rabbit IgG (H + L) (Abclonal, AS014, 1:5000).

### Statistical Analysis

The study used a paired sample design. A paired *t* test was used to compare lactylation levels and capacitation parameters between pre- and post-capacitation samples. The normality of the paired differences was verified using the Shapiro–Wilk test for both datasets (*p* > 0.05). A *p*-value<0.05 was considered statistically significant. All statistical analyses were carried out using R software (version 4.5.2).

## Results

### Post-translational Lactylation Landscapes During Sperm Capacitation Identified by Lactylation Proteomics

To confirm successful capacitation, we examined tyrosine phosphorylation levels, sperm motility, and acrosome reaction rates in non-capacitated and capacitated sperm samples, all of which verified that capacitation was successfully established (Supplemental Fig. S1). Subsequently, we conducted lactylation profiling of human sperm before and after capacitation to identify key lactylated proteins and sites. As shown in Figure 1*A*, we identified 20,726 peptides, 9841 modified peptides, 9697 quantifiable sites, and ultimately 2156 modified proteins (2030 quantifiable). To comprehensively understand the functional characteristics of the identified proteins, we performed systematic functional annotations including GO, Protein domains, KEGG pathways, COG/KOG (23, 24) classification, Subcellular localization, Reactome, WikiPathways, HallMark (25), and TFs (26, 27). The annotatihumanon categories with corresponding protein/site counts were displayed in Figure 1*B*. Furthermore, we identified thousands lactylation sites in current study. Understanding the BP underlying these modifications represents a crucial direction in current research. We therefore analyzed amino acid residue features surrounding modification sites (motif analysis), examining peptide sequences comprising 10 upstream and downstream amino acids for each identified site. Figure 1*C* presented the frequency variation scores of adjacent amino acids through heatmap visualization, demonstrated higher occurrence of alanine, isoleucine, and valine around modification sites.

**Fig. 1:**
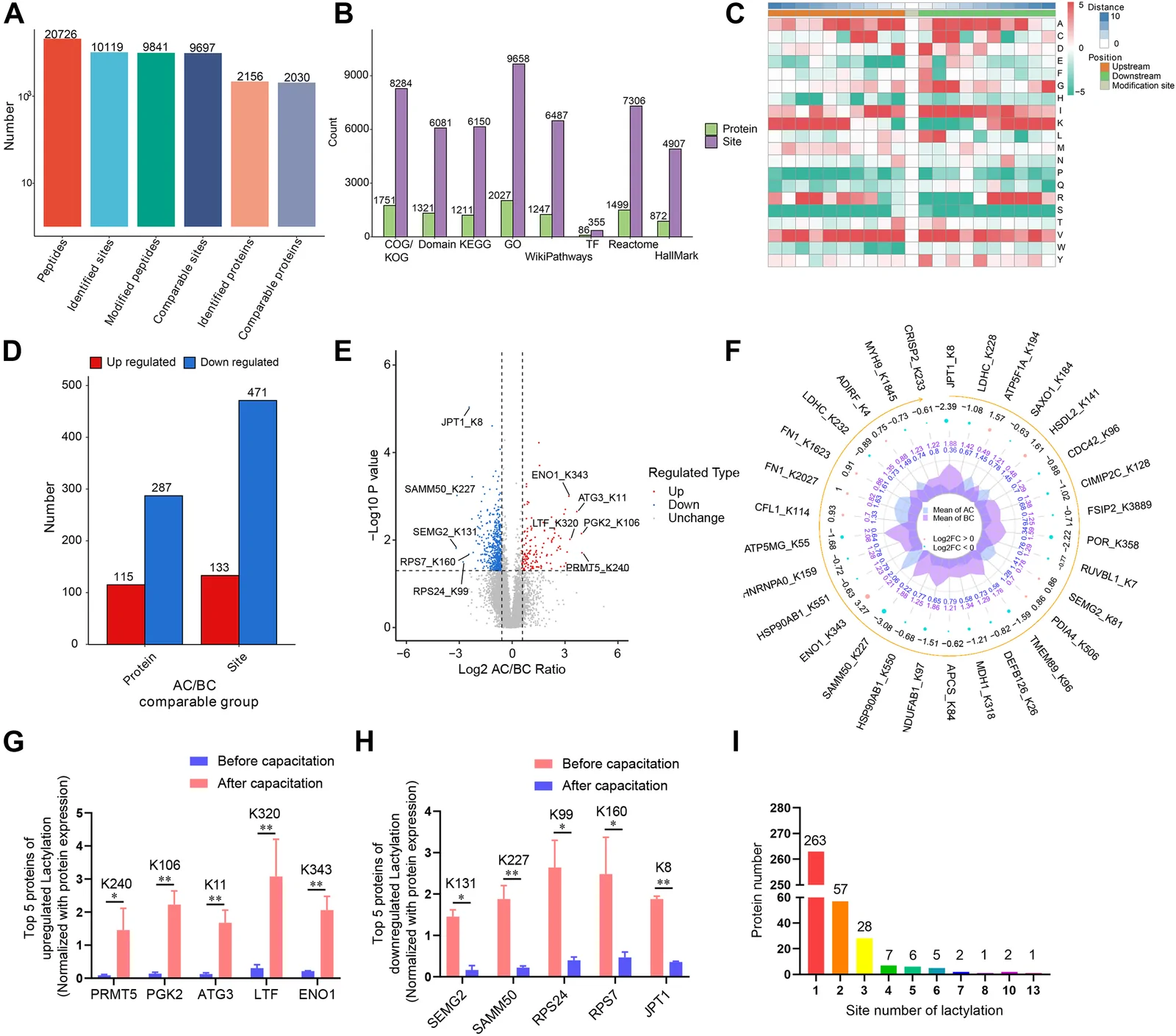
**Panoramic view of sperm protein lactylation before and after capacitation.***A*, the number of identified peptides, sites, proteins, modified peptides, comparable sites and proteins. *B*, counts of annotated proteins and sites by GO, Domain、KEGG、COG/KOG、Reactome、WikiPathways、HallMark and TF. *C*, frequency of amino acid occurrence near modification sites. *D*, number of regulated proteins/sites with lactylation modification before and after sperm capacitation. *p* < 0.05, Fold Chang>1.5. *E*, the volcano plot displays differentially modified proteins and sites. The top five upregulated and downregulated proteins/sites were marked. *p* < 0.05, Fold Chang>1.5. *F*, the radar chart visually displays the upregulated and downregulated proteins, sites, and multiples. *G* and *H*, the relative quantitation of the five upregulated and five downregulated sites with the most significant differential modifications. Data are presented as the Mean ± S.E.M., N = 3 in each group, ∗*p* < 0.05, ∗∗*p* < 0.01. *I*, differential site number of proteins with lactylation modification.

Further analysis of differential lactylation (protein expression-normalized, *p* < 0.05, Fold Change>1.5) revealed 115 upregulated proteins/133 sites and 287 downregulated proteins/471 sites post-capacitation (Fig. 1*D*), with downregulated modifications significantly outnumbering upregulated ones. Figure 1*E* displayed these differential proteins/sites in a volcano plot, with the top five upregulated sites being PRMT5(K240), PGK2(K106), ATG3(K11), LTF(K320), and ENO1(K343), and the top five downregulated sites being SEMG2(K131), SAMM50(K227), RPS24(K99), RPS7(K160), and JPT1(K8). Relative quantification of these 10 sites was shown in Figure 1, *G* and *H*. Figure 1*F* visualized the protein/site quantities and fold changes through radar chart. These results demonstrate substantial lactylation remodeling during capacitation, suggesting its critical regulatory role in this process.

Quantitative analysis of lactylation sites distribution revealed distinct modification patterns: 263 proteins exhibited single-site modification, followed by progressively decreasing numbers with multiple sites (57 at two sites, 28 at three, etc.), with three proteins showing exceptional hyper-lactylation (>10 sites) (Fig. 1*I*, *p* < 0.05, Fold Change>1.5). Intriguingly, expression-level analysis demonstrated differential regulation patterns: 79.1% (91/115) of proteins with upregulated lactylation maintained stable expression versus 74.6% (214/287) in downregulated group (Supplemental Fig. S2). The identification of 30 dual-regulation proteins (22 stable, three downregulated) suggests sophisticated site-specific regulatory mechanisms governing sperm protein function.

### Association of Protein Lactylation Changes With Energy Metabolism Pathways During Human Sperm Capacitation

We conducted GO secondary classification, KEGG pathway analysis, COG/KOG classification, and subcellular localization analysis on the proteins with differential lactylation modification. In the GO secondary classification, the BP with the highest enrichment was organic substance metabolic process, the Cellular Component (CC) category was intracellular anatomical structure, and the Molecular Function (MF) was protein binding (Supplemental Fig. S3*A*). KEGG pathway analysis revealed the most enriched Cellular Process being transport and catabolism, the predominant Genetic Information Processing category being folding, sorting and degradation, and the primary Metabolic Processes involving carbohydrate, amino acid, and energy metabolism (Supplemental Fig. S3*B*). COG/KOG classification showed the highest enrichment in Cellular Processes and Signaling for PTM, protein turnover, chaperones, in Information Storage and Processing for translation, ribosomal structure and biogenesis, and in Metabolism for energy production and conversion (Supplemental Fig. S3). Supplemental Fig. S2, *D*–*F* displayed the subcellular localization of differentially modified proteins (D), downregulated proteins (E), and upregulated proteins (F). The majority of differential proteins were distributed in the cytoplasm, followed by nuclear proteins and extracellular proteins as the second and third respectively, with mitochondrial proteins ranking fourth, and all of them exceeding 10% proportion. Supplemental Fig. S4 visually presented pathway relationships (first circle), categories (second circle), statistical significance (third circle), protein quantities (fourth circle), and enrichment levels (fifth circle) from GO (A), KEGG (B), Reactome (C), and WikiPathways (D) enrichment analyses. Supplemental Fig. S4, *E* and *F* demonstrated domain enrichment in DnaJ, Proteasome, Cation transporter/ATPase, and N-terminus.

Subsequently, GO pathway analysis of proteins with reduced lactylation after capacitation revealed significant enrichment in BP including purine nucleotide biosynthetic process and ribonucleoside monophosphate metabolism, with marked enrichment in energy metabolism processes such as ATP metabolism, NAD metabolism, gluconeogenesis, and inner mitochondrial membrane organization (highlighted by red boxes) (Fig. 2*A*). CCs showed significant enrichment in sperm flagellar structures (“motile cilium”, “9+2 motile cilium”, “sperm flagellum”, “sperm principal piece”, “sperm fibrous sheath”) and mitochondrial energy metabolism components (“mitochondrial proton-transporting ATP synthase complex”) (Fig. 2*B*). MFs were predominantly enriched in ATP hydrolysis activity, acetyl-CoA C-acyltransferase activity, and L-lactate dehydrogenase activity (Fig. 2*C*). Similarly, KEGG pathway analysis identified enrichment in energy metabolism pathways including OXPHOS, glycolysis/gluconeogenesis, and pyruvate metabolism (Fig. 2*D*). Reactome analysis demonstrated enrichment in mitochondrial protein degradation, mitochondrial biogenesis, tricarboxylic acid (TCA) cycle, respiratory electron transport, chemiosmotic ATP synthesis, and gluconeogenesis (Fig. 2*E*). WikiPathways analysis corroborated these findings through enrichment of glycolysis/gluconeogenesis, aerobic glycolysis, mitochondrial electron transport chain, and krebs cycle regulation (Fig. 2*F*). Consistent conclusions across multiple analytical approaches indicate that reduced lactylation modifications in OXPHOS and glycolysis during sperm capacitation plays a decisive role in energy metabolism control.

**Fig. 2:**
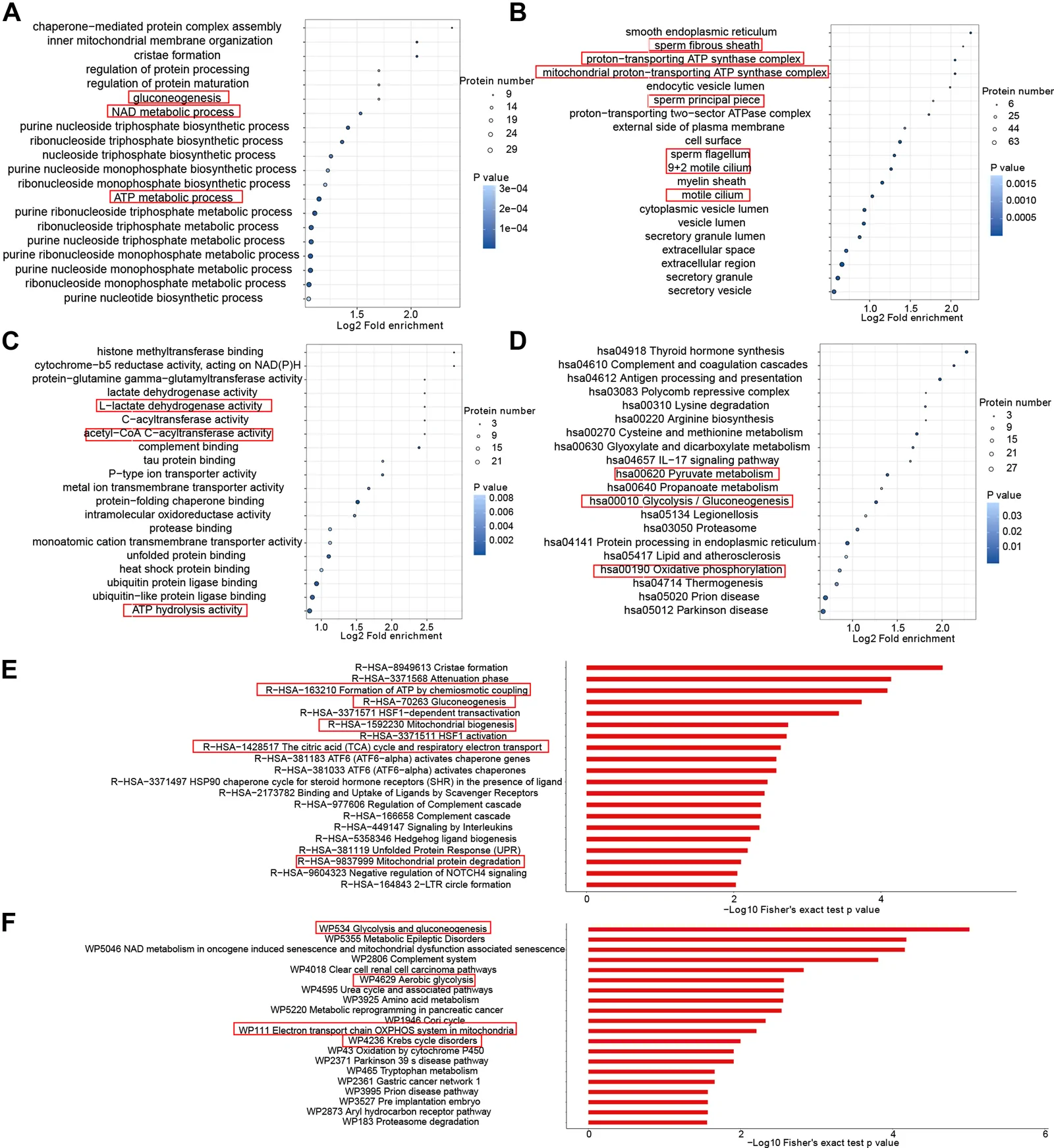
**Downregulation of lactylation plays a key role in energy metabolism during sperm capacitation.***A–C*, the GO enrichment analysis of proteins with downregulated lactylation modification including BP (*A*), CC (*B*) and MF (*C*). *D*–*F*, the KEGG (*D*), Reactome (*E*) and WikiPathways (*F*) analysis of proteins with downregulated lactylation modification. The red boxes indicated the enrichment in energy metabolism processes.

Furthermore, Supplemental Fig. S5 presented the pathway enrichment analysis of proteins with upregulated lactylation. In GO analysis, significantly enriched BP included regulation of DNA-templated transcription, regulation of RNA biosynthetic process, positive regulation of protein metabolic process, negative regulation of apoptotic process, and regulation of endopeptidase activity, which were predominantly associated with RNA synthesis and protein metabolism (Supplemental Fig. S5*A*). The enriched CCs comprised extracellular region, extracellular space, secretory granule lumen, cell cortex, and vesicle lumen (Supplemental Fig. S5*B*). MFs were primarily enriched in small molecule binding, nucleoside phosphate binding, nucleotide binding, ribonucleotide binding, and purine ribonucleotide binding (Supplemental Fig. S5*C*). The KEGG (Supplemental Fig. S5*D*), Reactome (Supplemental Fig. S5*E*), and WikiPathways (Supplemental Fig. S5*F*) analyses, visualized through cirplot and barplot representations respectively, revealed enrichment in fundamental cellular BP and pathways including RNA degradation, cellular stress responses, unfolded protein response, and PI3K-Akt signaling pathway. Notably, these pathway analyses did not demonstrate significant pathway specificity.

### Lactylation-Downregulation of Respiratory Chain Complex Proteins May Play a Key Role in Regulating Sperm Energy Patterns During Capacitation

To compare functional differences among proteins with varying lactylation fold changes, we divided proteins into four subgroups (Q1-Q4) based on fold change ratios (FC = AC/BC; Q1: <0.5, Q2: 0.5–0.667, Q3: 1.5–2.0, Q4: >2.0). Each subgroup underwent GO, KEGG, Reactome, and WikiPathways enrichment analyses followed by functional clustering. Similar to conventional pathway enrichment results, Q1 and Q2 proteins showed enrichment in energy metabolism-related BP through GO (Fig. 3, *A*–*C*), KEGG (Fig. 3*D*), Reactome (Supplemental Fig. S6*A*), and WikiPathways (Supplemental Fig. S6*B*) analyses, including OXPHOS, citrate cycle, glycolysis, gluconeogenesis, pyruvate metabolism, lactate biosynthesis/degradation, ATP synthesis, and respiratory electron transport. The CCs enriched across Q1-Q4 included sperm flagella, "9+2" motile cilium, sperm principal piece, sperm fibrous sheath, mitochondrial membrane, mitochondrial inner membrane, and mitochondrial proton-transporting ATP synthase complex, which were structures crucial for sperm energy metabolism and motility. GSEA analysis yielded significant findings: proteins in the lactate biosynthetic process from pyruvate and ATP synthesis-coupled electron transport were enriched at the bottom of the ranking list in GO analysis (Supplemental Fig. S7), and OXPHOS and pyruvate metabolism-related proteins showed similar patterns in KEGG analysis (Supplemental Fig. S8), indicating an overall downregulation trend. Subsequently, we systematically analyzed the functions, sperm expression patterns, and sperm localization of all differentially lactylated proteins related to OXPHOS/glycolysis using Human Protein Atlas (28, 29) and UniProt databases (detailed in Supplemental Table S1).

**Fig. 3:**
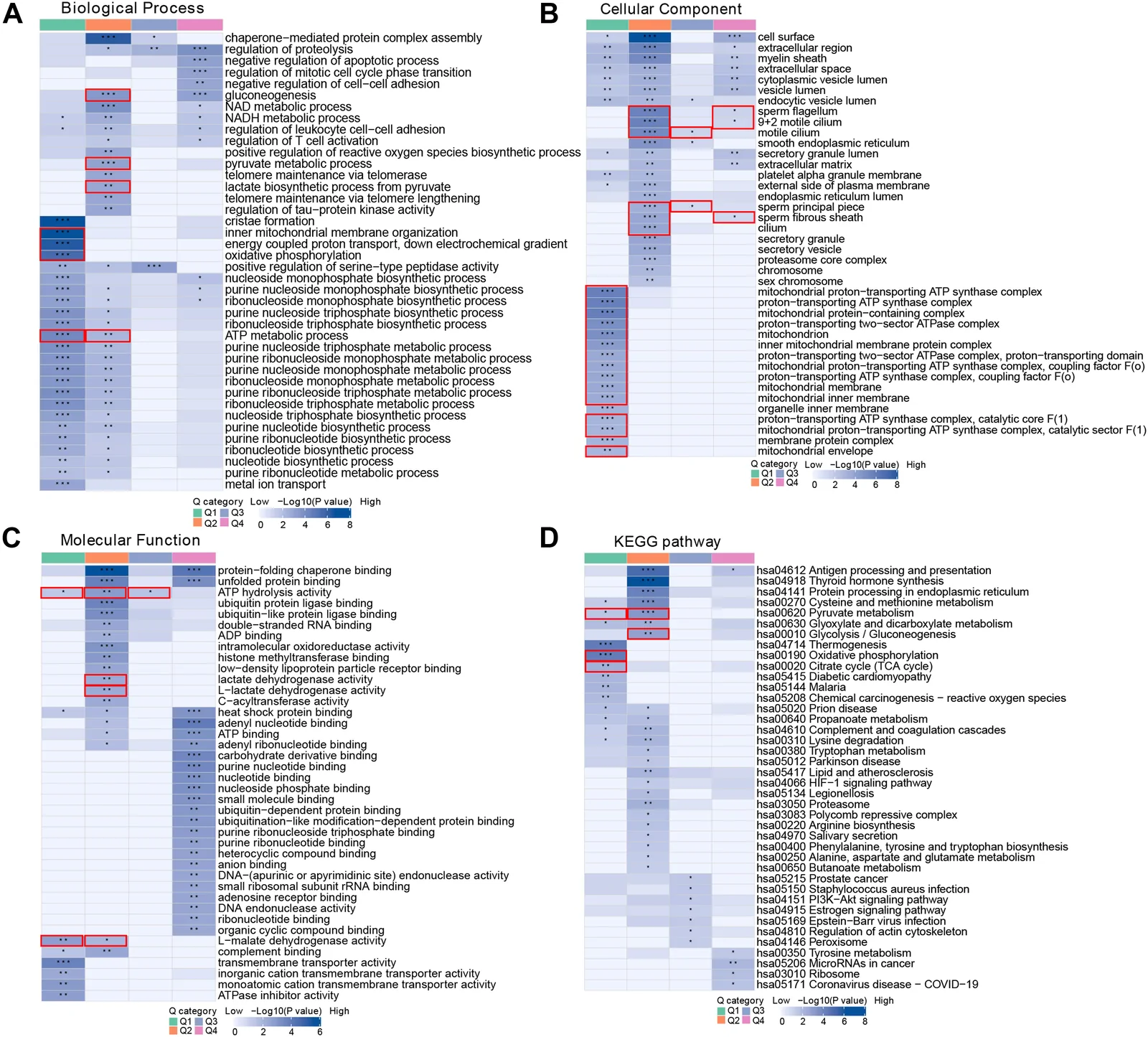
**Functional clustering analysis of proteins with different fold changes.** Enrichment analysis of GO (*A*–*C*) and KEGG (*D*) was performed for proteins of Q1-Q4. Fold change = AC/BC, Q1: <0.5, Q2: 0.5 to 0.667, Q3: 1.5 to 2.0, Q4: >2.0. *p* < 0.05.

Given the marked downregulation of lactylation in respiratory chain proteins, we visualized the OXPHOS pathway. As shown in Figure 4*A*, red fills indicate upregulated lactylation, blue fills denote downregulated ones, and yellow fills represent nodes containing both. Downregulated proteins included NDUFAB1 (Complex I), SDHB (Complex II), COR1/QCR2 (Complex III), COX6C (Complex IV), and ATP3/ATP5E/ATP5O/ATP5F1/ATP5I/ATP5L/ATP8 (Complex V). ATP5A1 (Complex V) was upregulated, while ATP5H showed mixed regulation. Figure 4*B* displayed quantitative lactylation changes of these proteins. These findings suggested that the reduced lactylation of respiratory chain complexes during sperm capacitation may critically regulate metabolic mode switching.

**Fig. 4:**
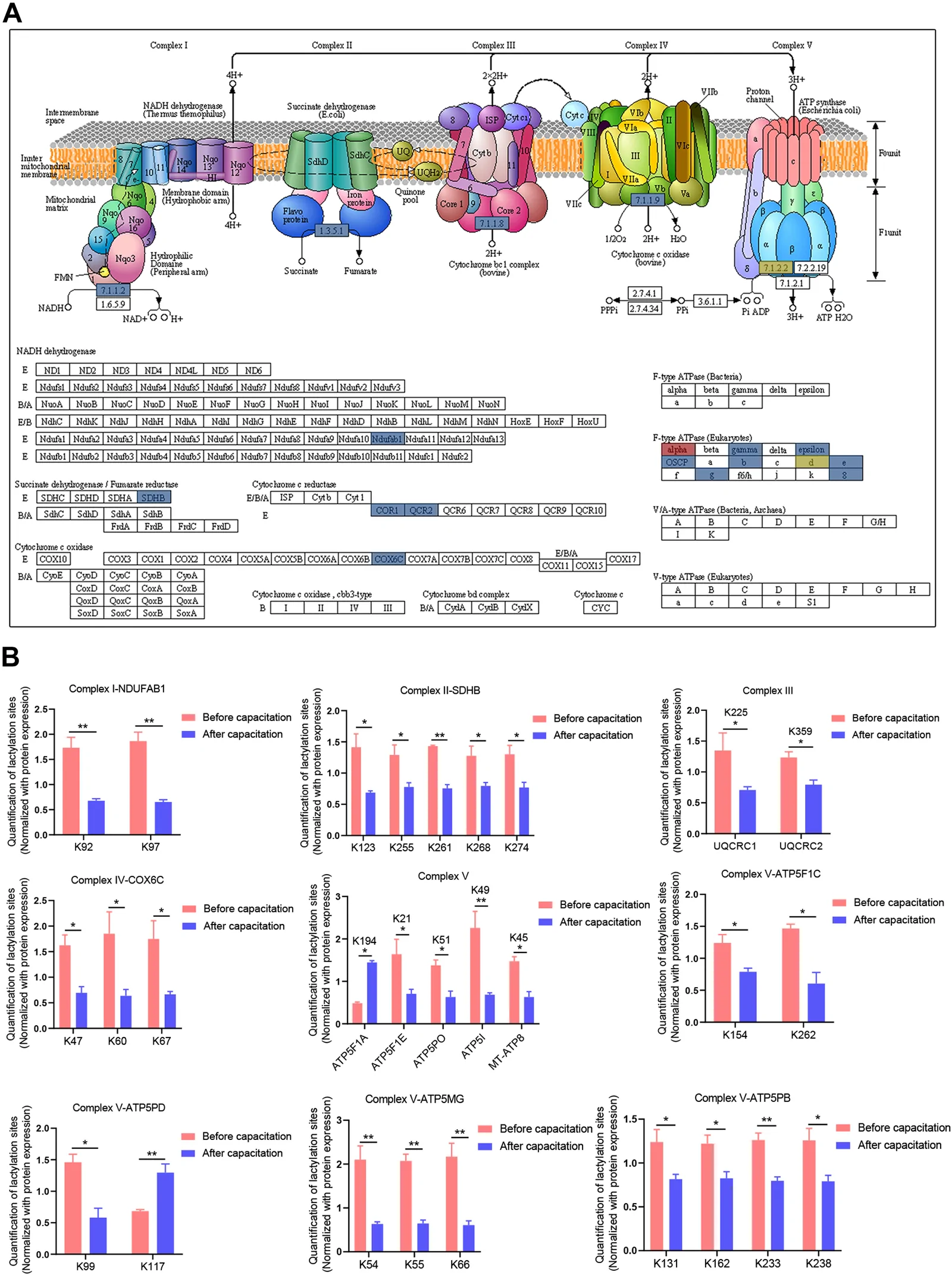
**Downregulated lactylation of respiratory chain complex proteins was observed after capacitation.***A*, visualization of proteins with differential lactylation modification in respiratory chain complex. *Red fills* indicate upregulation, *blue fills* denote downregulation, and *yellow fills* represent nodes containing both. *B*, quantitative analysis of lactylation modification of respiratory chain proteins. Data are presented as the Mean ± S.E.M., N = 3 in each group, ∗*p* < 0.05, ∗∗*p* < 0.01.

### Lactylation-Driven Regulation of Capacitation Associated Biological Pocesses in Spermatozoa

Our pathway analysis uncovered lactylation’ s novel regulatory roles in key fertilization events. Database mining identified 21 fertilization-related proteins (5.2% of 402 differential targets) with dynamic lactylation changes post-capacitation: three upregulated targets (acrosome formation/head remodeling) versus 18 downregulated (acrosome reaction/zona penetration) (Supplemental Table S2). Notably, nine capacitation regulators showed lactylation shifts: three increasing (fibrous sheath integrity, cAMP-dependent kinase modulation, sperm motility regulation) and six decreasing (Na+/K + ATPase regulation, serine phosphorylation, acrosome reaction, sperm hyperactivation). These findings propose a previously unrecognized regulatory axis where lactylation modulates energy metabolism during sperm-egg fusion and hyperactivation, representing a paradigm shift in understanding post-translational regulation of fertilization.

Mechanistic exploration extended lactylation’ s influence to fundamental biological processes: (1) metabolic regulation (fatty acid β-oxidation), (2) genetic information flow (RNA splicing/translation), (3) protein homeostasis (degradation/chaperoning), and (4) cytoskeletal dynamics (microtubule regulation) (Supplemental Table S3). The most striking discovery concerns flagellar microtubule lactylation. While glutamylation/glycylation are established motility regulators, our finding of lactylated microtubule proteins introduces a novel regulatory layer potentially governing flagellar hyperactivation. This unprecedented mechanism, distinct from known PTM pathways, opens new research directions in male reproductive biology.

### Identification of Potential Sperm Regulatory Proteins of Lactylation

Lactate participates in lactylation modification through two main pathways regulated by the "Writer-Eraser" dynamic balance: First, *via* lactoyl-CoA as a donor to form covalent modifications on lysine residues; Second, through direct catalysis by lactate transferases such as alanyl-tRNA synthetase (AARS1/2) that combine lactate with ATP (30). Recent studies have reported AARS1/2 acting as lactate sensors in mammalian cells to promote protein lactylation by functioning as lactate transferases (30). Meanwhile, HDAC1-3 (5), 6 (31) and SIRT1-3 (32) have been previously identified as key delactylation enzymes that dynamically regulate this process. We therefore examined expression changes of these proteins during capacitation. Interestingly, AARS1, SIRT2, SIRT3, HDAC1, HDAC2, and HDAC6 were all detected in sperm proteins, with SIRT3 showing 1.6-fold upregulation while other proteins remained unchanged (Fig. 5, *A*–*F*). Besides, no significant difference in SIRT3 lactylation was observed between pre- and post-capacitation samples (Fig. 5*G*). Although AARS1 expression showed no significant alteration (Fig. 5*A*), its lactylation level at K650 site reduced (Fig. 5*H*). Whether this modification affects AARS1′ s lactyltransferase activity remains unknown, potentially representing a feedback regulation mechanism to reduced lactate levels.

**Fig. 5:**
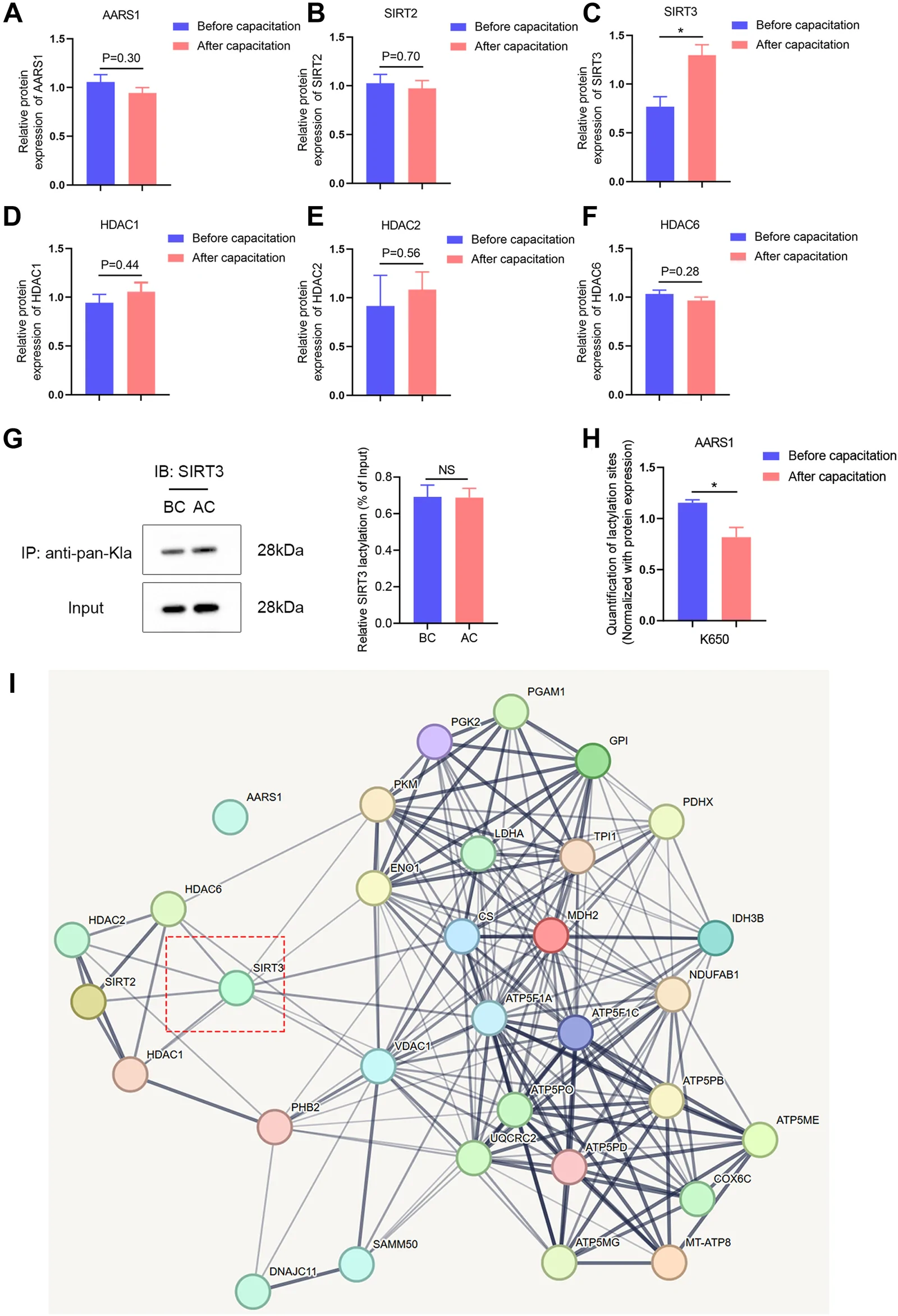
**Identification of potential lactylation regulatory proteins during sperm capacitation.***A*–*F*, relative quantitative analysis of AARS1, SIRT2, SIRT3, HDAC1, HDAC2, and HDAC6 before and after sperm capacitation. *G*, the lactylation level of SIRT3 showed no significance difference before and after capacitation (BC and AC). The right panel represents the quantitative densitometric analysis of the corresponding protein bands. Data are presented as the mean ± S.E.M., N = 4 per group, NS, no significance. *H*, the lactylation level at K650 site of AARS1 was reduced after sperm capacitation. *I*, potential interactions between AARS1, SIRT2, SIRT3, HDAC1, HDAC2, HDAC6 and energy metabolism-related differentially lactylated proteins (including OXPHOS and glycolysis) were analysed using String.

We therefore selected these six candidates (AARS1, SIRT2, SIRT3, HDAC1, HDAC2, and HDAC6) for STRING analysis, aiming to explore their potential interactions with our identified energy metabolism-related differentially lactylated proteins (OXPHOS and glycolysis enzymes). Figure 5*I* demonstrated potential interactions between them. Protein-protein interactions were analyzed using STRING (https://string-db.org/). SIRT3 occupies a central regulatory position, showing potential interactions with VDAC1, ATP5PO, ATP5F1A, CS, ENO1, and PKM. Flexible docking between SIRT3 and VDAC1, ATP5PO, ATP5F1A, CS, ENO1, and PKM were performed on the HADDOCK webserver. The first complex model with the lowest HADDOCK score was chosen to be the final model (Fig. 6). Subsequently, Co-IP assays further revealed the interactions between SIRT3 and those proteins (Fig. 6, *A*–*F*, right panel).

**Fig. 6:**
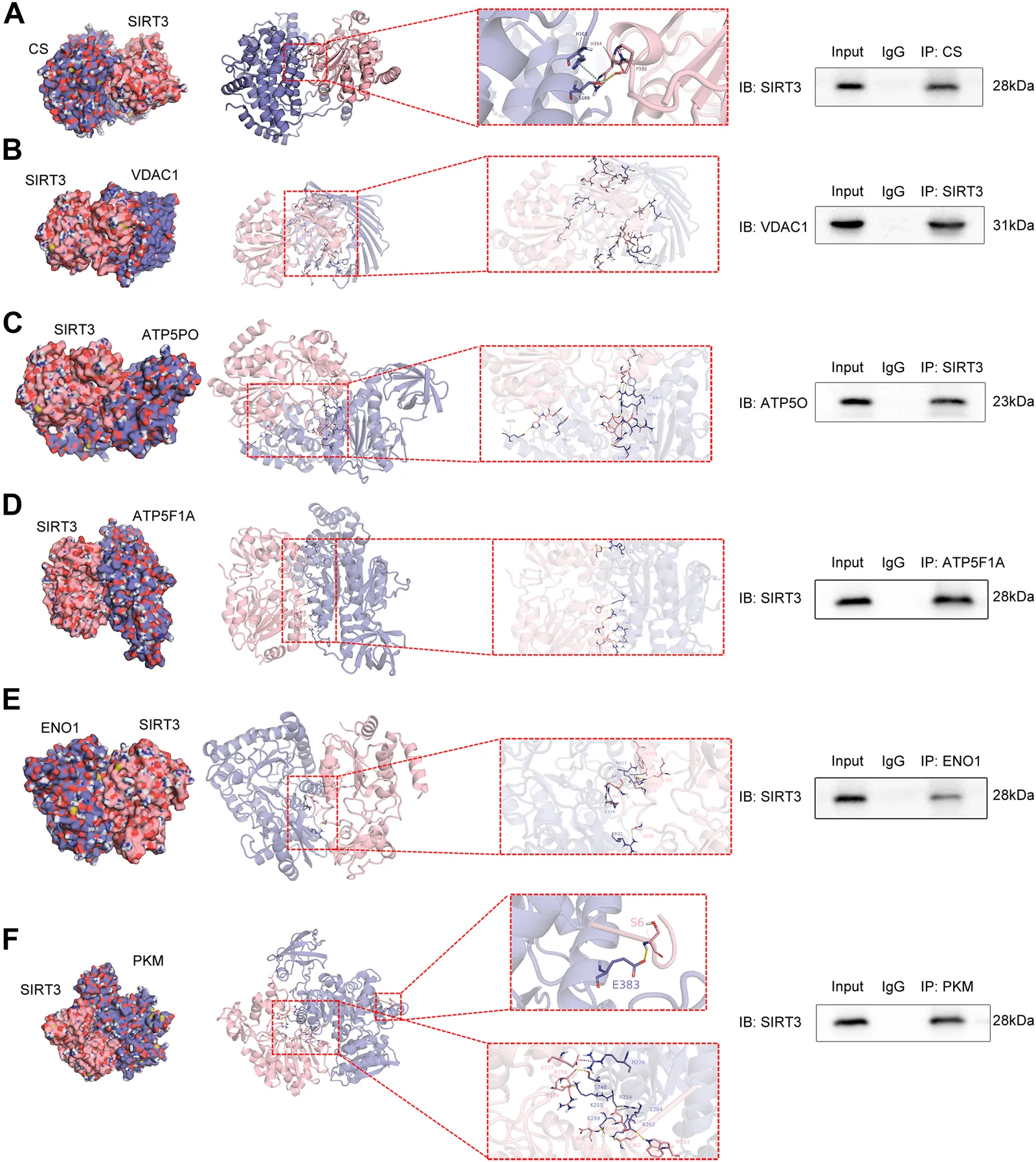
**Structural models of SIRT3 in complex with its predicted interacting partners.** Three-dimensional structures of SIRT3, CS, VDAC1, ATP5PO, ATP5F1A, ENO1, and PKM were retrieved from the AlphaFold Protein Structure Database (https://alphafold.com/). Molecular docking between SIRT3 and each predicted target protein was performed using the HADDOCK web server (https://rascar.science.uu.nl/haddock2.4/). For each SIRT3–target pair, the complex model with the lowest HADDOCK score was selected as the representative binding conformation. The right panels showed the interactions between SIRT3 and these proteins, as determined by Co-IP and Western blotting.

SIRT3 has been reported as a key delactylase, and our lactylomic data revealed its upregulation during capacitation. To explore whether SIRT3 physically associates with mitochondrial respiratory chain proteins, we performed Co-IP assays using an anti-SIRT3 antibody, followed by immunoblotting with antibodies against subunits of the five mitochondrial complexes. As shown in Figure 7*A*, SIRT3 was found to interact with NDUFAB1 (Complex I), SDHB (Complex II), UQCRC1 (Complex III), COX6C (Complex IV), and ATP5L (Complex V), suggesting a broad association with mitochondrial respiratory machinery. To preliminarily investigate the functional relevance of SIRT3-mediated delactylation in capacitation, we treated spermatozoa with honokiol, a SIRT3 agonist, at concentrations of 0.1, 1, and 5 μM during *in vitro* capacitation. Interestingly, only the 0.1 μM concentration significantly enhanced both total and progressive sperm motility (Fig. 7*B*), while higher concentrations showed no notable effect. Additionally, 0.1 μM honokiol treatment reduced global protein lactylation levels (Fig. 7*C*), consistent with SIRT3 activation promoting delactylation. Collectively, these findings suggest that SIRT3 may play a potential role in regulating sperm capacitation, possibly through its interaction with mitochondrial proteins and modulation of lactylation, although further mechanistic investigation is warranted.

**Fig. 7:**
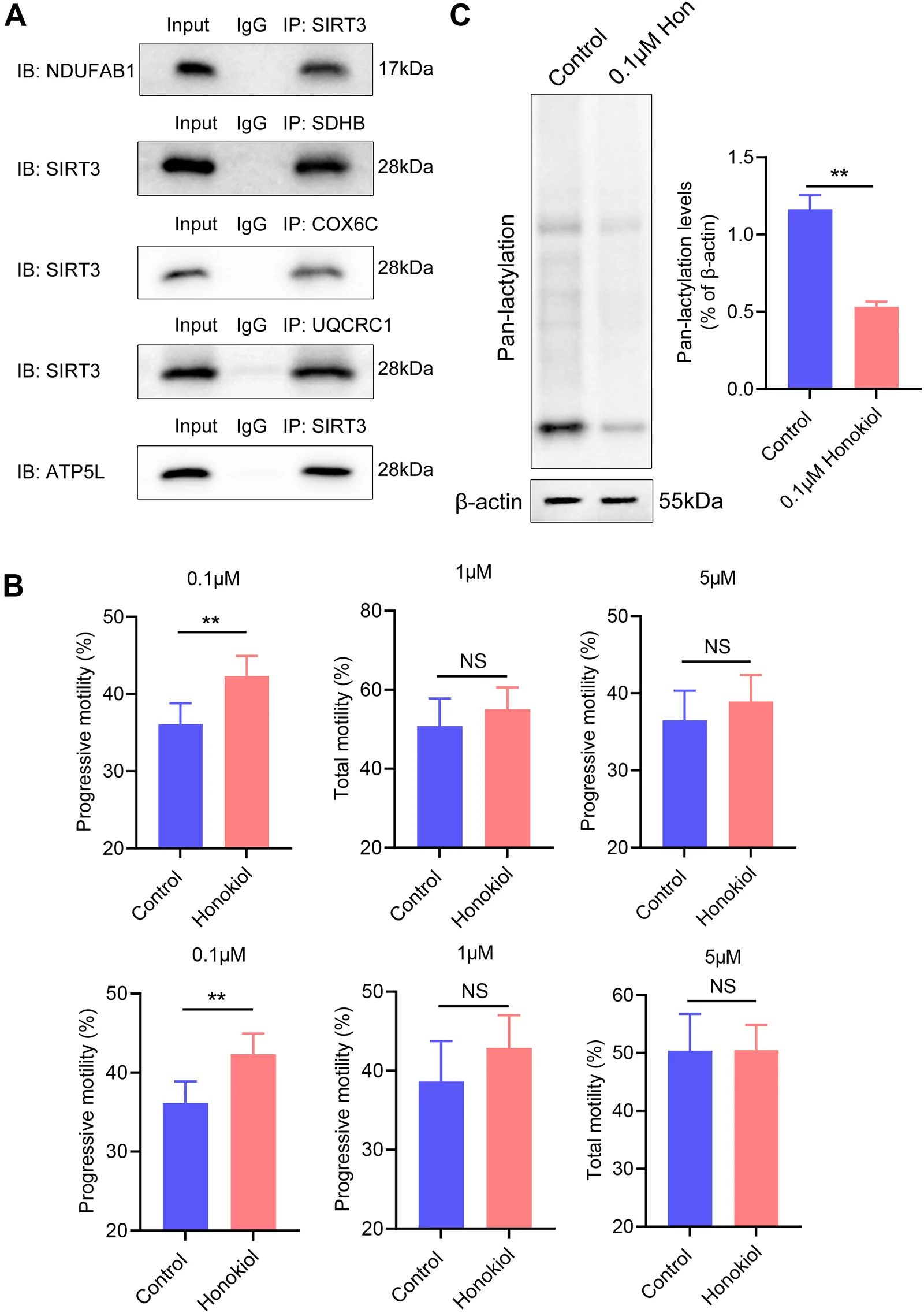
**SIRT3 interacts with mitochondrial respiratory chain proteins and its activation affects sperm motility and global lactylation.***A*, Co-IP was performed to reveal the interactions between SIRT3 and Mitochondrial respiratory chain proteins including NDUFAB1 (Complex I), SDHB (Complex II), UQCRC1 (Complex III), COX6C (Complex IV), and ATP5L (Complex V). *B*, sperm total motility and progressive motility were assessed after capacitation in the presence of honokiol (0.1, 1, and 5 μM). Data are presented as mean ± S.E.M., N = 8 per group. ∗∗*p* < 0.01, NS, no significance. *C*, pan-lactylation levels were detected by Western blot in spermatozoa treated with 0.1 μM honokiol or vehicle control. Data are presented as mean ± S.E.M., N = 4 per group. ∗∗*p* < 0.01.

## Discussion

Mammalian sperm require efficient ATP production to support progressive motility and hyperactivation, which are essential for capacitation and fertilization (33). Numerous studies demonstrate that glycolysis serves as the primary energy source for sperm motility (34, 35). As a glycolytic end-product, lactate plays crucial regulatory roles in sperm movement. Under physiological concentrations, lactate is oxidized to pyruvate via LDHC, subsequently converted to acetyl-CoA by pyruvate dehydrogenase for TCA cycle entry to promote ATP generation. The resulting NADH further enhances OXPHOS and ATP production (16). Emerging evidence reveals lactate can enter mitochondria to activate electron transport chains, enabling cells to optimize lactate utilization while reducing glucose dependence (36). These findings establish lactate not as metabolic waste, but as a master regulator of cellular energetics, critically governing sperm energy metabolism and male fertility. Lactylation modification, as a groundbreaking discovery in life sciences, holds profound multidimensional significance. Its “metabolism-epigenetics-function” triad not only expands understanding of metabolite functions, but provides novel perspectives for disease mechanism elucidation and therapeutic development. Serving as the core substrate, lactate dynamically modulates intracellular PTM networks through its metabolic flux, bridging energy metabolism with epigenetic regulation. The pathogenic roles of lactylation have been validated across diseases, becoming crucial breakthroughs in mechanism research. AARS1/2 can sense intracellular lactate fluctuations to regulate lactylation levels of cGAS, p53, etc., influencing DNA repair, tumorigenesis and immune evasion (30, 37). However, its functions in male reproduction, particularly sperm energy metabolism, remain completely unexplored. Addressing this knowledge gap holds landmark significance for unveiling lactylation’ s regulatory mechanisms in sperm bioenergetics.

This study employed high-resolution lactylome profiling to systematically map dynamic protein lactylation landscapes during human sperm capacitation. Post-capacitation analysis identified 115 upregulated proteins with 133 modified sites and 287 downregulated proteins with 471 modified sites, showing significant predominance of downregulated targets (*p* < 0.05, Fold change>1.5). The substantial quantity of differential lactylation events suggests its critical regulatory role in capacitation. Further analysis revealed site-specific modification patterns: 263 proteins exhibited single-site modification, followed by 57 (two sites), 28 (three sites), with three hyper-lactylated proteins showing >10 modified sites. These findings imply that mono-lactylation may predominantly drive functional modulation in sperm proteins. Lactylation regulates protein function through multiple mechanisms: molecular interaction modulation, stability control, enzymatic activity adjustment, subcellular localization, histone lactylation-mediated epigenetic regulation, and crosstalk with other PTMs (1, 8, 9, 38, 39, 40). In this study, 91/115 (79.1%) upregulated lactylated proteins maintained stable expression versus 214/287 (74.6%) in downregulated group. This expression-independent regulation implies lactylation predominantly induces conformational/activity changes during capacitation. Intriguingly, 30 proteins showed bidirectional site modifications (22 expression-stable, three downregulated), indicating complex multi-site regulatory networks governing sperm protein function.

Pathway enrichment analysis of upregulated lactylated proteins revealed significant associations with DNA-templated transcription regulation, RNA biosynthetic processes, and positive regulation of protein metabolism. During spermiogenesis, protamine replacement mediates sperm chromatin compaction (41, 42). While mature sperm are transcriptionally inert, post-testicular maturation processes (epididymal transit, capacitation, acrosome reaction) require stored RNA-derived protein synthesis (43). The upregulated lactylation of transcriptional/translational regulators suggests a novel epigenetic mechanism coordinating late-stage functional protein synthesis during fertilization preparation. Downregulated lactylated proteins were enriched in energy metabolism pathways (ATP metabolic, NAD metabolic, OXPHOS, Glycolysis/Gluconeogenesis, Pyruvate metabolism, L-lactate dehydrogenase activity). Recent studies show mitochondrial AARS2 acts as lactyltransferase whose degradation via PHD2-mediated proline hydroxylation is oxygen-sensitive (17). Hypoxia-induced AARS2 accumulation increases PDHA1 (K336) and CPT2 (K457/8) lactylation, suppressing OXPHOS by limiting acetyl-CoA flux. SIRT3 reverses these modifications to activate OXPHOS. Our observed delactylation of mitochondrial complex proteins likely enhances enzymatic activity to meet capacitation’ s energy demands. Intriguingly, glycolytic enzyme lactylation downregulation suggests cross-talk between OXPHOS and glycolysis. LDHC (testis-specific LDH) showed increased expression with decreased lactylation post-capacitation, potentially forming a feedback loop: delactylation enhances LDHC activity, increased lactate-pyruvate conversion, reduced lactate levels, further delactylation. And the generated pyruvate enters the TCA cycle to promote sperm ATP production. This metabolic-epigenetic coupling facilitates TCA cycle-driven ATP surge for fertilization.

The regulation of lactylation relies on the dynamic "Writer-Eraser" balance. Recent studies have identified AARS1/2 as lactate-sensing proteins functioning as lactyltransferases to promote protein lactylation in mammalian cells (30). Meanwhile, HDAC1-3 (5), 6 (31) and SIRT1-3 (32) have been confirmed as key delactylation enzymes that dynamically regulate this process through lactyl group removal. We therefore investigated expression changes of these Writer-Eraser proteins during capacitation. Intriguingly, AARS1, SIRT2, SIRT3, HDAC1, HDAC2, and HDAC6 were all detected in sperm proteins via mass spectrometry. Among the candidate lactylation regulators examined, SIRT3 was the only one showing a 1.6-fold upregulation at the protein level during capacitation, suggesting its potential involvement in delactylation regulation. To functionally test this possibility, we treated spermatozoa with the SIRT3 agonist honokiol (0.1 μM) during capacitation. This treatment significantly reduced global lactylation levels and enhanced both total and progressive sperm motility, providing initial evidence supporting a role for SIRT3 in delactylation regulation during sperm capacitation. Besides, although AARS1 expression remained unchanged, we observed decreased lactylation at its K650 site. Whether this modification affects AARS1′ s lactyltransferase activity remains unknown, potentially representing a feedback mechanism in response to reduced lactate levels. Current mechanistic investigations are ongoing.

This study observed significant enrichment of differentially lactylated proteins in biological processes including fatty acid β-oxidation, RNA splicing/translation, protein degradation, chaperone processing, and microtubule dynamics during capacitation, demonstrating the broad regulatory scope of lactylation. As a pioneering lactylation mapping study, we fill critical knowledge gaps in sperm biology and establish molecular foundations for future investigations. However, limitations exist: our *in vitro* capacitation system may incompletely replicate physiological hypoxia conditions, where oxygen tension fluctuations could influence lactate availability and subsequent lactylation dynamics. Furthermore, we acknowledge that in terms of regulatory mechanisms, these findings are preliminary, and further experiments—such as using SIRT3 inhibitors, knockdown/overexpression approaches, or lactylation site-specific mutants—are needed to firmly establish the causal role of specific lactylation events in regulating capacitation. This work provides direct evidence for the "metabolism-epigenetics-function" triad, expanding lactate’ s biological significance and offering new diagnostic/therapeutic avenues for male infertility. Future studies should employ spatiotemporal lactylation mapping and single-cell multi-omics to decipher germ cell-specific regulatory mechanisms. Pharmacological modulation of lactyltransferases/delactylases may emerge as innovative strategies to rectify sperm metabolic dysfunction.

## Ethical Approval and Participation Consent

The study was performed in accordance with the guidelines of the Declaration of Helsinki and approved by the Medical Ethics Committee of Sichuan Provincial Woman’ s and Children’ s Hospital (20,240,903–357), and all patients provided signed informed consents.

## Data Availability

The mass spectrometry proteomics data have been deposited to the ProteomeXchange Consortium (https://proteomecentral.proteomexchange.org/cgi/GetDataset?ID=PXD079545) *via* the iProX partner repository with the dataset identifier PXD079545. Detailed information regarding the identified and quantified proteins, including accession numbers, peptide counts, and relative quantitation data, is provided in Supplemental Tables S4 and S5.

## Supplemental data

This article contains supplemental data.

## Conflict of interest

The authors declare that they have no conflicts of interest with the contents of this article.
