# Supplementary material for: Comparative Lactylomic Profiling Unveils Dynamic Reprogramming of Post-translational Lactylation Landscapes During Human Sperm Capacitation
Source: Mol Cell Proteomics. 2026 Sep 8;25(10):101657. doi: 10.1016/j.mcpro.2026.101657 (PMC13693420; doi:10.1016/j.mcpro.2026.101657)
Supplement: Supplementary file 1 [file mmc1.zip › Supplementary Figures.docx]

**Supplementary Figures**

**Figure S1. Confirmation of successful sperm capacitation.** (A-B) Total motility (A) and progressive motility (B) of spermatozoa were increased after capacitation. (C) Tyrosine phosphorylation levels of sperm proteins were elevated after capacitation. The right panel represents the quantitative densitometric analysis of the corresponding protein bands. (D-E) Coomassie brilliant blue staining of spermatozoa was performed to evaluate acrosomal status. Red arrows indicate acrosome-intact spermatozoa with deeply stained acrosomes; blue arrows indicate acrosome-reacted spermatozoa with lightly stained or unstained acrosomes. A total of 100 spermatozoa were counted per sample. Data are presented as the mean ± S.E.M., N=6 per group, *P < 0.05, **P < 0.01. BC, before capacitation; AC, after capacitation.

**Figure S2. Venn Diagram of sperm proteins identified in current research****Figure S3.** GO (A) secondary classification, KEGG pathway analysis (B), COG/KOG classification (C), and subcellular localization analysis (D-F) on the proteins with differential lactylation modification

**Figure S4.** Pathway relationships (first circle), categories (second circle), statistical significance (third circle), protein quantities (fourth circle), and enrichment levels (fifth circle) from GO (A), KEGG (B), Reactome (C), and WikiPathways (D) enrichment analyses. Domain enrichment in DnaJ, Proteasome, Cation transporter/ATPase, and N-terminus (E-F).

**Figure S5. The pathway enrichment analysis of proteins with upregulated lactylation during sperm capacitation.** The enriched BP (A), CCs (B) and MFs (C) of GO analysis. The KEGG (D), Reactome (E), and WikiPathways (4F) analyses, visualized through cirplot and barplot representations respectively.**Figure S6. Q1 and Q2 proteins showed enrichment in energy metabolism-related BP through Reactome (A) and WikiPathways (B) analyses.**

**Figure S7. GSEA summary of GO terms.**

**Figure S8. GSEA summary of KEGG terms.**

**Figure S9. Validation of lactylation levels of LDHA, MDH2, and ODF1 before and after capacitation by co-immunoprecipitation.** Sperm lysates from non-capacitated (BC) and capacitated (AC) samples were subjected to immunoprecipitation (IP) using an anti-pan-lactyl-lysine (pan-Kla) antibody, followed by immunoblotting (IB) with antibodies against LDHA, MDH2, and ODF1, respectively. Total cell lysates (Input) were used as loading controls. The right panels represent the quantitative densitometric analysis of the corresponding protein bands. Data are presented as the mean ± S.E.M., N=4 per group, **P < 0.01. BC, before capacitation; AC, after capacitation.
