# Supplementary material for: Comparative Lactylomic Profiling Unveils Dynamic Reprogramming of Post-translational Lactylation Landscapes During Human Sperm Capacitation
Source: Mol Cell Proteomics. 2026 Sep 8;25(10):101657. doi: 10.1016/j.mcpro.2026.101657 (PMC13693420; doi:10.1016/j.mcpro.2026.101657)
Supplement: Supplementary file 1 [file mmc1.zip › Table S1.docx]

| **Table S1. The functions, sperm expression patterns, and sperm localization of all differentially lactylated proteins related to oxidative phosphorylation/glycolysis** | | | | | | |
| --- | --- | --- | --- | --- | --- | --- |
| Biological Process | Protein  expression | Lactylation quantification | Protein  Name | High  expression  in sperm | Sperm localization | Function |
| Oxidative phosphorylation | Normal | Up | CS | NO | Mitochondrion | Key enzyme of the tricarboxylic acid cycle |
|  |  |  | VDAC1 |  |  | Facilitates the exchange of metabolites across the outer mitochondrial membrane |
|  |  |  | ATP5F1A |  |  | Mitochondrial membrane ATP synthase |
|  |  | Down | PHB2 |  |  | Stabilizes mitochondrial respiratory enzymes |
|  |  |  | IDH3B |  |  | Isocitrate dehydrogenase subunit β |
|  |  |  | UQCRC2 |  |  | Component of the ubiquinol-cytochrome c oxidoreductase |
|  |  |  | MDH2 |  |  | Malate dehydrogenase |
|  |  |  | ATP5PB |  |  | Mitochondrial membrane ATP synthase |
|  |  |  | ATP5F1C |  |  |  |
|  |  |  | MT-ATP8 |  |  |  |
|  |  |  | ATP5PO |  |  |  |
|  |  |  | ATP5MK |  |  |  |
|  |  |  | PDHX |  | Unclear | Component of mitochondrial pyruvate dehydrogenase |
|  |  |  | SAMM50 |  | Mitochondrion, Acrosome | Maintenance of the structure of mitochondrial cristae |
|  |  | Up/Down | MDH1 |  | Mitochondrion | Catalyzes the NAD/NADH-dependent, reversible oxidation of malate to oxaloacetate |
|  |  |  | DNAJC11 |  | Unclear | Required for mitochondrial inner membrane organization |
|  |  |  | ATP5PD |  | Mitochondrion | Mitochondrial membrae ATP synthase |
|  | Up | Down | NDUFAB1 |  | Mitochondrion | Transfer of electrons from NADH to the respiratory chain |
|  |  |  | COX6C |  |  | Component of the cytochrome c oxidase |
|  |  |  | ATP5MG |  |  | Mitochondrial membrane ATP synthase |
|  |  |  | ATP5ME |  |  |  |
| Glycolysis | Normal | Down | LDHA | NO | Principal piece | Stereospecifically interconverts simultaneously and pyruvate and lactate |
|  |  |  | LDHB |  | Unclear |  |
|  |  |  | GPI | YES | Mitochondrion | Catalyzes the conversion of glucose-6-phosphate to fructose-6-phosphate |
|  |  |  | PKM |  | Principal piece | Catalyzes the final rate-limiting step of glycolysis |
|  |  |  | PGAM1 | NO | Unclear | Catalyzes the interconversion of 2-phosphoglycerate and 3-phosphoglyceratea crucial step in glycolysis |
|  |  |  | TPI1 |  | Principal piece | Catalyzes the interconversion between dihydroxyacetone phosphate and D-glyceraldehyde-3-phosphate |
|  |  | Up/Down | ENO1 | NO | Middle piece, principal piece | Catalyzes the conversion of 2-phosphoglycerate to phosphoenolpyruvate |
|  |  |  | PGK2 | YES | Middle piece, principal piece | Catalyzes the reversible conversion of 1,3-bisphosphoglycerate to 3-phosphoglycerate |
