# Supplementary material for: Comparative Lactylomic Profiling Unveils Dynamic Reprogramming of Post-translational Lactylation Landscapes During Human Sperm Capacitation
Source: Mol Cell Proteomics. 2026 Sep 8;25(10):101657. doi: 10.1016/j.mcpro.2026.101657 (PMC13693420; doi:10.1016/j.mcpro.2026.101657)
Supplement: Supplementary file 1 [file mmc1.zip › Table S2.docx]

| **Table S2. The functions, sperm expression patterns, and sperm localization of all differentially lactylated proteins related to fertilization and capacitation** | | | | | |
| --- | --- | --- | --- | --- | --- |
| Biological Process | Lactylation quantification | Gene  Name | High  expression  in sperm | Sperm localization | Function |
| Fertilization | Up | RAB2A | NO | Acrosome | Acrosome biosynthesis |
|  |  | DPY19L2 | YES |  | Head elongation and acrosome formation |
|  |  | SPACDR |  |  | Acrosome formation and nucleus shaping |
|  | Down | ACR | YES |  | Lysis of the zona pellucida |
|  |  | CD46 | NO |  | Sperm-oocyte fusion |
|  |  | CD55 | YES |  |  |
|  |  | LGALS3 | NO |  |  |
|  |  | UBA1 |  |  |  |
|  |  | ERP29 |  | Head and middle piece |  |
|  |  | PDIA3 |  | Equatorial segment and anterior flagella |  |
|  |  | ACRV1 | YES | Acrosome | Sperm-zona binding or penetration |
|  |  | SPAM1 |  |  | Sperm-egg adhesion |
|  |  | CDC42 | NO |  | Fertilization |
|  |  | TCP11 | YES | Unclear | Sperm capacitation and acrosome reactions |
|  |  | CCDC136 |  | Acrosome | Acrosome formation |
|  |  | ZPBP |  |  | Acrosome compaction and sperm morphogenesis |
|  |  | DEFB126 | NO | Acrosome and middle piece | Penetrate the cumulus matrix and bind to the zona pellucida |
|  |  | SPESP1 | YES | Acrosome | Sperm-oocyte fusion |
|  |  | GLIPR1L1 |  |  |  |
|  |  | LYZL4 | NO | Acrosome and principal piece | Fertilization |
|  |  | PRSS54 | YES | Acrosome | Acrosomal granule localization and sperm head morphogenesis |
| Sperm capacitation | Up | PRKAR1A | NO | Middle piece | Regulatory subunit of the cAMP-dependent protein kinases |
|  |  | AKAP4 | YES | Principal piece | Regulation of sperm motility |
|  |  | ROPN1L |  | Flagella | Fibrous sheath integrity and sperm motility |
|  | Down | AKAP3 | YES | Acrosome, middle and principal piece | Spermatozoa motility and maintenance of acrosome structure |
|  |  | ATP1A1 | NO | Unclear | Na+/K+ATPase regulates sperm capacitation |
|  |  | DLD |  | Acrosome, middle piece | Involved in the acrosome reaction and the hyperactivation of spermatazoa during capacitation |
|  |  | CDC37 |  | Middle and principal piece | Regulates serine phosphorylation in human sperm capacitation |
|  |  | PARK7 |  | Acrosome, flagella | Preventing sperm from undergoing premature capacitation |
|  |  | ELSPBP1 |  | Unclear | Promote sperm capacitation |
