# Supplementary material for: Comparative Lactylomic Profiling Unveils Dynamic Reprogramming of Post-translational Lactylation Landscapes During Human Sperm Capacitation
Source: Mol Cell Proteomics. 2026 Sep 8;25(10):101657. doi: 10.1016/j.mcpro.2026.101657 (PMC13693420; doi:10.1016/j.mcpro.2026.101657)
Supplement: Supplementary file 1 [file mmc1.zip › Table S3.docx]

**Table S3. Categorization of differentially lactylated proteins by fundamental biological processes**

| Biological Process | Protein | Sperm Specific Expression | Location in Sperm | Protein Expression | Lactylation |
| --- | --- | --- | --- | --- | --- |
| Fatty acid β-oxidation | ACAT1 | No | Mitochondrion | No significance | Down regulation |
| Fatty acid β-oxidation | HADHA | No | Mitochondrion | No significance | Down regulation |
| Fatty acid β-oxidation | ACAA2 | No | Mitochondrion | No significance | Down regulation |
| Fatty acid β-oxidation | DECR1 | No | Mitochondrion | No significance | Down regulation |
| Fatty acid β-oxidation | HADHA | No | Mitochondrion | No significance | Down regulation |
| RNA splicing and protein translation | RPLP0 | No | Unclear | No significance | Up regulation |
| RNA splicing and protein translation | EIF2S1 | No | Unclear | No significance | Up regulation |
| RNA splicing and protein translation | EIF4E | No | Unclear | No significance | Up regulation |
| RNA splicing and protein translation | RPS2 | No | Unclear | No significance | Up regulation |
| RNA splicing and protein translation | EIF2S2 | No | Unclear | No significance | Up regulation |
| RNA splicing and protein translation | RPS3 | No | Unclear | No significance | Up regulation |
| RNA splicing and protein translation | RPS19 | No | Unclear | No significance | Up regulation |
| RNA splicing and protein translation | RPS5 | No | Unclear | No significance | Up regulation |
| RNA splicing and protein translation | RPL26 | No | Unclear | No significance | Up regulation |
| RNA splicing and protein translation | HNRNPK | No | Unclear | No significance | Up regulation |
| RNA splicing and protein translation | RPS13 | No | Unclear | No significance | Up regulation |
| RNA splicing and protein translation | HNRNPU | No | Unclear | No significance | Up regulation |
| RNA splicing and protein translation | HSP90AA1 | No | Unclear | No significance | Down regulation |
| RNA splicing and protein translation | EIF2S3 | No | Unclear | No significance | Down regulation |
| RNA splicing and protein translation | RPL5 | No | Unclear | No significance | Down regulation |
| RNA splicing and protein translation | EIF1AX | No | Unclear | No significance | Down regulation |
| RNA splicing and protein translation | METAP2 | No | Unclear | No significance | Down regulation |
| RNA splicing and protein translation | HNRNPA3 | No | Unclear | No significance | Down regulation |
| RNA splicing and protein translation | EIF4A1 | No | Unclear | No significance | Down regulation |
| RNA splicing and protein translation | DHX9 | No | Unclear | No significance | Down regulation |
| RNA splicing and protein translation | HNRNPA0 | No | Unclear | No significance | Down regulation |
| RNA splicing and protein translation | EIF3J | No | Unclear | Up regulation | Down regulation |
| RNA splicing and protein translation | RPL7 | No | Unclear | Up regulation | Down regulation |
| RNA splicing and protein translation | RPS7 | No | Unclear | Up regulation | Down regulation |
| RNA splicing and protein translation | RPS24 | No | Unclear | Up regulation | Down regulation |
| RNA splicing and protein translation | EEF1A1 | No | Unclear | No significance | Up/Down regulation |
| Protein degradation | PSMC2 | No | Unclear | No significance | Up regulation |
| Protein degradation | USP14 | No | Sperm nuclear membrane, head | No significance | Up regulation |
| Protein degradation | USP4 | No | Unclear | No significance | Up regulation |
| Protein degradation | PCNP | No | Unclear | No significance | Up regulation |
| Protein degradation | UFD1 | No | Unclear | No significance | Up regulation |
| Protein degradation | ATG3 | No | Unclear | No significance | Up regulation |
| Protein degradation | PSMD11 | No | Unclear | No significance | Down regulation |
| Protein degradation | UBA1 | No | Acrosome | No significance | Down regulation |
| Protein degradation | PSMA3 | No | Acrosome, flagella | No significance | Down regulation |
| Protein degradation | PSMA4 | No | Acrosome | No significance | Down regulation |
| Protein degradation | PSMB4 | No | Unclear | No significance | Down regulation |
| Protein degradation | TPP2 | No | Unclear | No significance | Down regulation |
| Protein degradation | PSMD8 | No | Unclear | No significance | Down regulation |
| Protein degradation | PSMA6 | No | Unclear | No significance | Down regulation |
| Protein degradation | UBE2L3 | No | Unclear | No significance | Down regulation |
| Protein degradation | PSMD2 | No | Unclear | No significance | Down regulation |
| Protein degradation | CUL2 | No | Unclear | No significance | Down regulation |
| Protein degradation | PSMF1 | No | Unclear | No significance | Down regulation |
| Protein degradation | PSMD1 | No | Unclear | No significance | Down regulation |
| Protein degradation | PPP1R11 | No | Head, acrosome | No significance | Up/Down regulation |
| Protein degradation | CUL3 | No | Unclear | No significance | Up/Down regulation |
| Protein degradation | USP7 | No | Unclear | No significance | Up/Down regulation |
| Molecular chaperone proteins processed | HSPA8 | No | Manchette | No significance | Up regulation |
| Molecular chaperone proteins processed | TOMM34 | No | Mitochondrion | No significance | Up regulation |
| Molecular chaperone proteins processed | HYOU1 | No | Sperm plasma membrane | No significance | Up regulation |
| Molecular chaperone proteins processed | DNAJA2 | No | Unclear | No significance | Down regulation |
| Molecular chaperone proteins processed | P4HB | No | Unclear | No significance | Down regulation |
| Molecular chaperone proteins processed | HSP90AA1 | No | Unclear | No significance | Down regulation |
| Molecular chaperone proteins processed | HSPA1A | No | Unclear | No significance | Down regulation |
| Molecular chaperone proteins processed | CLU | No | Head | No significance | Down regulation |
| Molecular chaperone proteins processed | HSP90B1 | No | Acrosome | No significance | Down regulation |
| Molecular chaperone proteins processed | DNAJA1 | No | Unclear | No significance | Down regulation |
| Molecular chaperone proteins processed | STIP1 | No | Unclear | No significance | Down regulation |
| Molecular chaperone proteins processed | HSP90AB1 | No | Unclear | No significance | Up/Down regulation |
| Molecular chaperone proteins processed | HSPD1 | No | Unclear | No significance | Up/Down regulation |
| Molecular chaperone proteins processed | HSPA5 | No | Unclear | No significance | Up/Down regulation |
| Molecular chaperone proteins processed | HSPA9 | No | Acrosome and sperm tail | No significance | Up/Down regulation |
| Molecular chaperone proteins processed | HSPA4L | Yes | Mitochondrion | No significance | Up/Down regulation |
| Microtubule dynamic protein | DYNC1H1 | No | Unclear | No significance | Up regulation |
| Microtubule dynamic protein | PFN1 | No | Unclear | No significance | Up regulation |
| Microtubule dynamic protein | CFL1 | No | Unclear | No significance | Up regulation |
| Microtubule dynamic protein | TUBB4B | No | flagella | No significance | Up regulation |
| Microtubule dynamic protein | MYO6 | No | Unclear | No significance | Up regulation |
| Microtubule dynamic protein | CLMN | No | Unclear | No significance | Up regulation |
| Microtubule dynamic protein | WDR1 | No | Unclear | No significance | Down regulation |
| Microtubule dynamic protein | STMN1 | No | Unclear | No significance | Down regulation |
| Microtubule dynamic protein | MSN | No | Unclear | No significance | Down regulation |
| Microtubule dynamic protein | MYH9 | No | Unclear | No significance | Down regulation |
| Microtubule dynamic protein | FKBP4 | No | Unclear | No significance | Down regulation |
| Microtubule dynamic protein | DCTN1 | No | Flagella | No significance | Down regulation |
| Microtubule dynamic protein | EFCAB6 | Yes | Flagellar axon microtubule | No significance | Down regulation |
| Microtubule dynamic protein | SAXO1 | Yes | Unclear | No significance | Down regulation |
| Microtubule dynamic protein | CFAP52 | No | Flagellar axon microtubule | No significance | Down regulation |
| Microtubule dynamic protein | EFHB | Yes | Flagellar axon microtubule | No significance | Down regulation |
| Microtubule dynamic protein | ENKUR | No | Acrosome, principal piece | No significance | Down regulation |
| Microtubule dynamic protein | DYNLRB2 | Yes | Unclear | No significance | Down regulation |
| Microtubule dynamic protein | DDI1 | Yes | Unclear | No significance | Down regulation |
| Microtubule dynamic protein | TEKT4 | Yes | Midpiece, principal piece | No significance | Down regulation |
| Microtubule dynamic protein | SPACA9 | Yes | Flagellar axon microtubule | No significance | Down regulation |
| Microtubule dynamic protein | TEKT5 | Yes | Flagella | No significance | Down regulation |
| Microtubule dynamic protein | ODAD4 | Yes | Flagellar axon microtubule | No significance | Down regulation |
| Microtubule dynamic protein | KIF2C | Yes | Unclear | No significance | Down regulation |
| Microtubule dynamic protein | DNAH17 | No | Flagella | No significance | Down regulation |
| Microtubule dynamic protein | CFAP210 | Yes | Flagellar axon microtubule | No significance | Down regulation |
| Microtubule dynamic protein | RSPH6A | Yes | Flagella | No significance | Down regulation |
| Microtubule dynamic protein | TMSB4X | No | Unclear | Up regulation | Down regulation |
| Microtubule dynamic protein | DNAH8 | Yes | Flagella | No significance | Up/Down regulation |
| Unclear | DNAJB8 | Yes | Unclear | No significance | Up regulation |
| Unclear | C10orf62 | Yes | Unclear | No significance | Up regulation |
| Unclear | SPACDR | Yes | Acrosome | No significance | Up regulation |
| Unclear | GARIN5B | Yes | Unclear | No significance | Up regulation |
| Unclear | IPO5 | Yes | Unclear | No significance | Down regulation |
| Unclear | TGM4 | Yes | Unclear | No significance | Down regulation |
| Unclear | EFHB | Yes | Unclear | No significance | Down regulation |
| Unclear | DYNLRB2 | Yes | Unclear | No significance | Down regulation |
| Unclear | SPACA9 | Yes | Unclear | No significance | Down regulation |
| Unclear | ODAD4 | Yes | Unclear | No significance | Down regulation |
| Unclear | KIF2C | Yes | Unclear | No significance | Down regulation |
| Unclear | ADAM29 | Yes | Unclear | No significance | Down regulation |
| Unclear | EFCAB3 | Yes | Unclear | No significance | Down regulation |
| Unclear | CFAP210 | Yes | Unclear | No significance | Down regulation |
| Unclear | IZUMO2 | Yes | Unclear | No significance | Down regulation |
| Unclear | SPATA31F1 | Yes | Unclear | No significance | Down regulation |
| Unclear | TEX30 | Yes | Unclear | No significance | Down regulation |
| Unclear | TMEM89 | Yes | Unclear | Up regulation | Down regulation |
| Unclear | KLK3 | Yes | Unclear | No significance | Up/Down regulation |
